# The dUTPase-related gene of bovine immunodeficiency virus is critical for viral replication, despite the lack of dUTPase activity of the encoded protein

**DOI:** 10.1186/1742-4690-11-60

**Published:** 2014-08-12

**Authors:** Nickolay Voronin, Eytan Herzig, Amnon Hizi

**Affiliations:** Department of Cell and Developmental Biology, Sackler School of Medicine, Tel Aviv University, Tel Aviv, 69978 Israel

**Keywords:** dUTPase, BIV, Lentiviruses, Retroviral replication

## Abstract

**Background:**

Deoxyuridine 5′-triphosphate nucleotide-hydrolases (dUTPases) are essential for maintaining low intra-cellular dUTP/dTTP ratios. Therefore, many viruses encode this enzyme to prevent dUTP incorporation into their genomes instead of dTTP. Among the lentiviruses, the non-primate viruses express dUTPases. In bovine immunodeficiency virus (BIV), the putative dUTPase protein is only 74 residues-long, compared to ~130 residues in other lentiviruses.

**Results:**

In this study, the recombinant BIV dUTPase, as well as infectious wild-type (WT) BIV virions, were shown to lack any detectable dUTPase activity. Controls of recombinant dUTPase from equine infectious anemia virus (EIAV) or of EIAV virions showed substantial dUTPase activities. To assess the importance of the dUTPase to BIV replication, we have generated virions of WT BIV or BIV with mutations in the dUTPase gene. The two mutant viral dUTPases were the double mutant D48E/N57S (in the putative enzyme active site and its vicinity) and a deletion of 36 residues. In dividing Cf2Th cells and under conditions where the WT virus was infectious and generated progeny virions, both mutant viruses were defective, as no progeny viruses were generated. Analyses of the integrated viral cDNA showed that cells infected with the mutant virions carry in their genomic DNA levels of integrated BIV DNA that are comparable to those in WT BIV-infected cells.

**Conclusions:**

The herby presented results show that the two BIV mutants with the modified dUTPase gene could infect cells, as viral cDNA was synthesized and integrated into the host cell DNA. However, no virions were generated by cells infected by these mutants. The most likely explanation is that either the integrated cDNA of the mutants is defective (due to potential multiple mutations, introduced during reverse-transcription) or that the original dUTPase mutations have led to severe blocks in viral replication at steps post integration. These results emphasize the importance of the dUTPase-related sequence to BIV replication, despite the lack of any detectable catalytic activity.

**Electronic supplementary material:**

The online version of this article (doi:10.1186/1742-4690-11-60) contains supplementary material, which is available to authorized users.

## Background

Cellular deoxyuridine 5′-triphosphate nucleotide-hydrolases (dUTPases) have an essential role in maintaining low cellular dUTP over dTTP ratios. This is done by catalyzing the hydrolysis of dUTP into two products, the dTTP precursor-dUMP, and pyrophosphate (PPi) [1–3]. Consequently, the dUTPase activities lead to low dUTP/dTTP ratios; thus preventing deoxyuridine misincorporation into DNA, since most DNA polymerases can utilize dUTP instead of dTTP for DNA synthesis. To keep uracils out of DNA, it is likely that most free-living organisms, as well as several DNA viruses (*e. g.,* herpesviruses and poxviruses) and some groups of retroviruses, encode dUTPases. Thus, this enzyme is essential for the viability of prokaryotes and eukaryotes and contributes to host range preferences and infectivity of viruses [1, 4].

**Figure 1:**
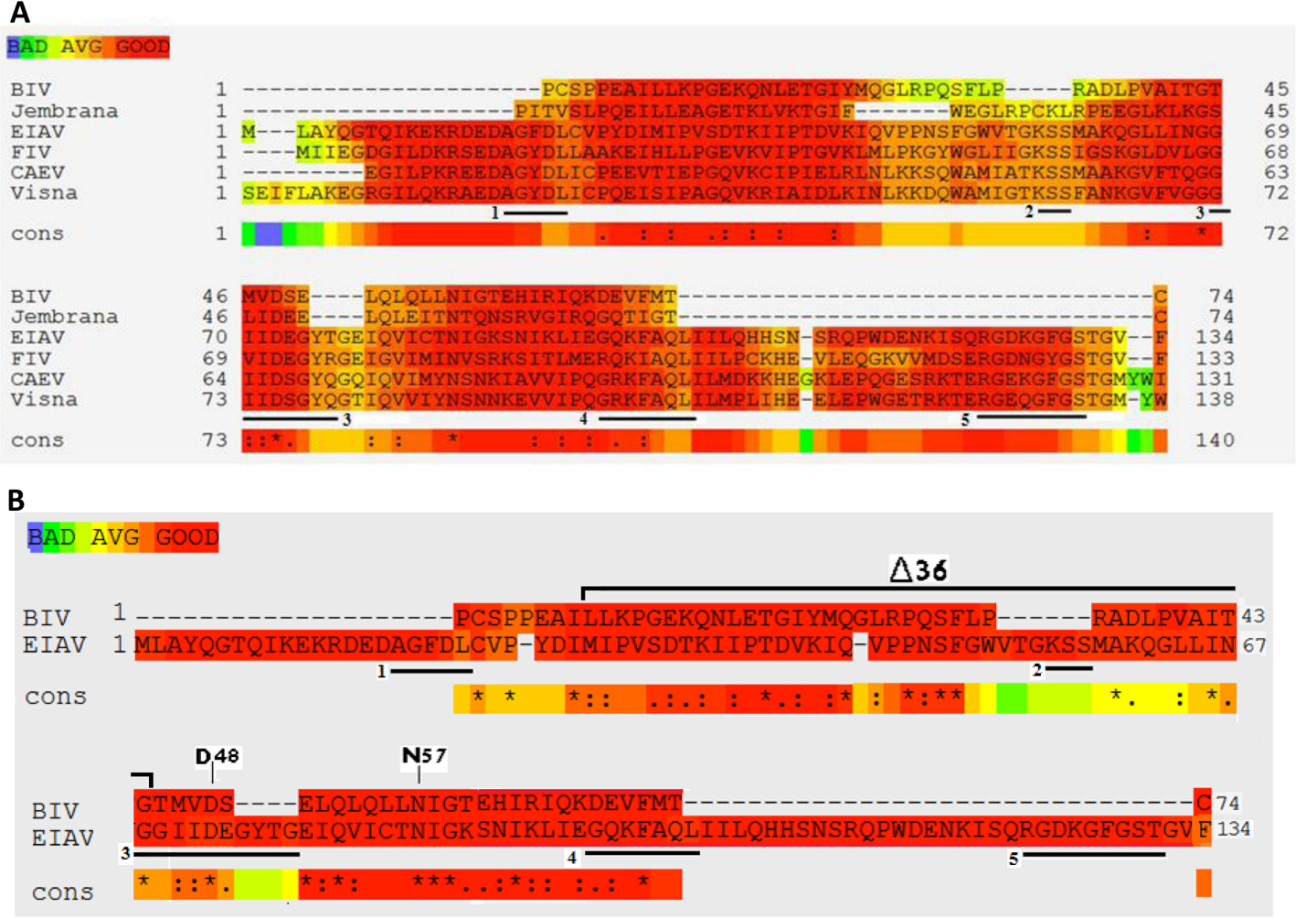
**Multiple amino acids sequence alignments of dUTPase proteins in different non-primate lentiviruses.** The sequence information was taken from The National Center for Biotechnology Information (EIAV dUTPase GI: 157830894; FIV dUTPase GI: 1942421; Visna Virus dUTPase GI:9626549; BIV dUTPase GI: 9626219; CAEV dUTPase GI: 266706151; Jembrana (JDV) dUTPase GI:733067). The figure was prepared using the T-COFFEE alignment tool (Version_9.03.r1318) [16, 17]. The locations of five conserved dUTPase motifs are underlined and numbered. **A**. Sequence alignments of non-primate lentiviruses dUTPase-encoding genes. **B**. BIV dUTPase *versus* EIAV dUTPase. The conserved mutated BIV dUTPase residues (D48E/N57S) and the Δ36 deletion mutation are marked above the sequences.

The replication of retroviruses heavily relies on the complex process of reverse transcription, the mechanism by which the retroviral plus-strand RNA genome is copied into double-stranded DNA [5]. This process is catalyzed entirely by the viral reverse transcriptase (RT) [6]. While synthesizing DNA, RT is capable of incorporating dUTP instead of dTTP into the nascent DNA strands [7, 8]. This may explain why retroviral dUTPases are associated with increasing viral replication efficiency and a better fidelity of DNA synthesis, thus prevent deleterious mutations [9]. Cellular dUTPases are believed to be cell-cycle regulated with an elevated activity in dividing cells and a substantially lower activity in terminally-differentiated non-dividing cells, such as macrophages [10, 11]. Therefore, the endogenous pools of dUTP are supposed to be lower in dividing cells relative to the non-dividing ones. Consequently, retroviral dUTPases are advantageous for viral replication in non-dividing cells rather than in dividing cells [12–14]. Most dUTPases (including the retroviral ones) are homotrimers with five conserved sequence motifs [15], see also Figure 1A.

Despite sharing similar mechanisms of replication, only several groups of retroviruses encode dUTPases, while the others lack this enzyme. The dUTPase-expressing retroviruses are the beta-retroviruses and the non-primate lentiviruses, while other retroviruses (including the primate lentiviruses) lack a dUTPase-encoding gene. Among the non-primate lentiviruses, dUTPase-encoding sequences have been identified in feline immunodeficiency virus (FIV) [18], equine infectious anemia virus (EIAV) [12], caprine arthritis-encephalitis virus (CAEV) [13] and visna virus of sheep [19]. In addition, in the bovine lentiviruses, BIV and Jembrana disease virus (JDV), a homologous dUTPase-encoding gene is present [20, 21] (see also Figure 1A). Earlier studies revealed that retroviruses, encoding defective dUTPases, exhibit delayed replication kinetics in macrophages due to blocks in their replication cycle [12, 13]. In the case of visna virus, the dUTPase is also dispensable for the neuro-pathogenicity of the virus [19] and in the case of CAEV, dUTPase is necessary for the development of bilateral arthritis lesions in the carpus of the infected goats [22]. The dUTPase-defective viruses accumulate in their genome G to A transition mutations [9, 22]. Since primate lentiviruses lack dUTPases, it was suggested that, as an alternative, they recruit and package in the virions a cellular uracil DNA glycosylase, designated UNG [4, 23]. This enzyme removes uracils from the synthesized DNA, as part of the base-excision repair pathway. Although UNG and dUTPase are mechanistically different enzymes, they both participate in excluding uracils from DNA. Interestingly, the virion-associated UNG catalytic activity in human immunodeficiency virus type-1 (HIV-1) can be replaced by packaging into the virions a heterologous dUTPase from CAEV [24]. Collectively, these data suggest that, at least in the case of beta-retroviruses and some lentiviruses, uracil accumulation in the viral DNA can be detrimental to critical steps in retroviral life cycle. An interesting exception to this supposition was recently reported. It was found that HIV-1 could tolerate, or even benefits from, non-mutagenic uracil incorporation during intracellular reverse transcription [25]. Thus, in this case uracilation of the viral cDNA obstructs the strand transfer of the DNA ends by the integrase (IN), thereby inhibiting the suicidal auto-integration side pathway and facilitating the correct chromosomal integration and, hence, the viral replication.

All retroviruses encode three “classical” enzymes, namely, the protease (PR), RT and IN. All are indispensable for the viral life cycle [5]. Thus, dUTPase is the fourth and lesser studied retrovirus-encoded enzyme that is found only in several groups of these viruses. It is not completely clear why most retrovirus groups can replicate without having dUTPases, unless these viruses evade the need for such a viral-encoded enzyme by utilizing the cellular counterpart (as in the case of utilizing the UNG enzyme). Interestingly, and unlike the other retroviral enzymes, the dUTPase-encoding gene is situated in retroviruses in two entirely different locations, suggesting two different evolution pathways. In the prototype beta-retroviruses (namely, mouse mammary tumor virus-MMTV and Mason-Pfizer virus), the *gag-pol* genes encode three reading frames (*gag, pro and pol*) and the dUTPase protein is encoded by both the *gag* and *pro* reading frames. Therefore, the dUTPase protein is actually a trans-frame protein. To be more specific, the N-terminal segment (of ~95 residues) of this protein matches the C-terminal part of the Gag polyprotein; thus, this segment is precisely identical to the whole viral nucleocapsid (NC) protein (also known as p14) [26]. The C-terminus of the dUTPase is encoded by the 5′-end of the *pro* gene, ending next to the PR-encoding segment [27–29]. In these retroviruses, the dUTPase protein is about 250 residues long. In contrast, the *gag-pol* genes of lentiviruses are composed of only two reading frames (*gag* and *pol*). In the dUTPase-expressing lentiviruses, the encoding gene is located within the *pol* gene, between the RNase H (C-terminal) portion of the RT and the IN-encoding segment. Thus, it is conceivable that the dUTPase is a proteolytic product of the Gag-Pol polyprotein precursor. Moreover, the size of the lentivirus-associated dUTPases is about half (~130 residues) of the beta-retroviral dUTPases. For all the dUTPase-expressing retroviruses, there is sufficient evidence that, similar to the other three retroviral enzymes, the dUTPases are packaged into the viral particles in an enzymatically active form [12, 13, 22, 27, 29, 30].

The case of BIV and the closely-related JDV [31] is unique, though they follow the same pattern as the non-primate lentiviruses [32]. The putative dUTPase-encoding gene (that shows a limited homology to some of the conserved motifs in other dUTPases- see Figure 1) is also located between the RT and IN-encoding genes. However, the encoded polypeptide is substantially smaller than the ~130-residues long protein in the other lentiviruses, as it is estimated to be only 74 amino acids long (Figure 1) [20, 21]. This BIV protein lacks some of the five conserved motifs, characteristic of dUTPases. As far as we know, no other dUTPase-related protein, including all viral, prokaryotic or eukaryotic enzymes, is so small (ranging in most fully-documented cases between ~130 and ~300 residues). The BIV 74-residues-long polypeptide has not been extensively studied and it is not clear whether it can possess any significant dUTPase enzymatic activity. On the other hand, it is conceivable that this dUTPase-related peptide has a biological role, since it is conserved in both BIV and JDV (Figure 1) [31].

In order to characterize the BIV *pol* gene products, we have already studied the recombinantly-expressed RT and IN enzymes of BIV [20, 21]. While engineering the RT-encoding genome, and due to the low sequence homology at the C-terminus to other well studied RTs, we have expressed two RT versions. The first one was 546 residues-long and its C-terminus was located upstream and adjacent to the 74-residues long dUTPase-related peptide. The second, 620 residues-longer RT version, encompassed the dUTPase segment at its C-terminus (designated BIV RT-dU). Likewise, two BIV IN versions were generated and studied. The longer form, designated BIV dU-IN, was 353 residues-long, comprising at its N-terminus the 74 residue dUTPase-related polypeptide. The shorter version that lacks the dUTPase segment was 279 residues-long. In both cases of recombinant RT and IN forms, the longer, dUTPase-containing enzyme versions were slightly less active than the shorter versions. Therefore, it is likely from these *in vitro* studies with the recombinant proteins that the dUTPase-related segment is not essential for RT or IN activities.

The objective of the present study was to further study the dUTPase-related 74 residues-long peptide of BIV. First, we have expressed this short protein in bacteria, and after its purification, it was assayed to find whether it possesses any detectable dUTPase activity. In parallel, the other BIV Pol-derived enzymes that are fused to the dUTPase sequences (namely, BIV RT-dU and BIV dU-IN) were also tested for this activity. As a positive control, EIAV dUTPase was also expressed and assayed. None of the three BIV dUTPase-related proteins showed any detectable enzymatic activity (whilst the EIAV enzyme was highly active). Second, an infectious BIV clone was used to study the replication in cell cultures of the WT BIV and two of its dUTPase mutants. Unlike the WT virus, these two mutant viruses were fully defective in producing progeny virions, although the integration of viral cDNA into the host cell genome was apparently normal.

## Results and discussion

### Recombinant expression of the lentiviral dUTPases

In order to test whether the WT BIV dUTPase- related 74 residues segment has a detectable dUTPase enzymatic activity, we have expressed this recombinant polypeptide in bacteria with a six-histidine tag at its N-terminus. The protein was purified using the standard Ni-agarose (NTA) affinity columns, as was extensively used by us for purifying a variety of retroviral enzymes [20, 21, 33–37], (see Methods). As a positive lentiviral dUTPase control, which was previous confirmed to possess enzymatic activity both in virions [12] and as a recombinant protein [38], we have cloned, expressed in bacteria and purified the six-histidine tagged dUTPase of EIAV (see Methods). Sequence alignments of the different dUTPase proteins are shown in Figure 1. In addition, we have used the two versions of BIV dUTPase- the RT-dU and dU-IN that were fused to either RT or IN, respectively, as described earlier [20, 21].

**Figure 2:**
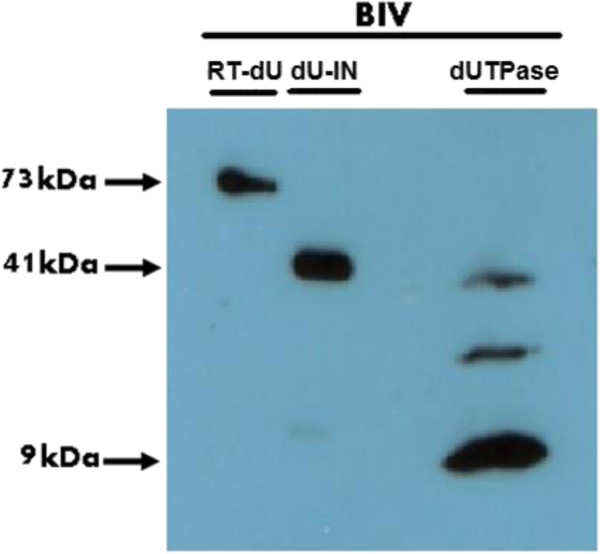
**Western analysis of the purified recombinant BIV dUTPase proteins.** The proteins were prepared and purified as described in Methods and in the text. The proteins were analyzed by 10% SDS/PAGE and the gels were then underwent western blot analysis. The blot was treated with 1:500 dilution of rabbit antiserum prepared against the dUTPase-derived peptide (see Methods). After blocking the serum-treated blot, it was treated with a 1:10,000 dilution of a secondary donkey HRP-conjugated anti-rabbit IgG antibody that was purchased from abcam.

Western analyses of the SDS-PAGE separated three recombinant BIV dUTPase-related proteins (performed with a rabbit anti-serum against a peptide with residues 48–67 of the BIV dUTPase) are shown in Figure 2. The purified WT dUTPase was composed of mostly of a ~9 kDa protein that is in good agreement with the expected molecular mass (74 residues of the dUTPase plus 8 additional residues at the N-terminal tail- see Methods). In addition, there were two minor anti-serum reactive proteins (with apparent MW values of roughly 28 kDa and 38 kDa). These minor bands (not detected in the RT-dU and IN-dU protein preparations) may result from some aggregation of the monomers (possibly to trimers and tetramers). The fused RT-dU and IN-dU proteins with MW of ~73 kDa and ~41 kDa, respectively, were already studied by us [20, 21]. In addition, we studied the purified 134 + 8 residues-long EIAV dUTPase that was shown to have a molecular mass value of ~15 kDa (data not shown).

### The dUTPase activity of the recombinant proteins

**Figure 3:**
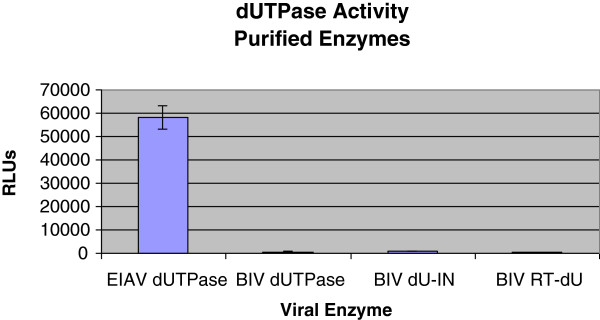
**The PPiLight pyrophosphate detection assay for the dUTPase enzymatic activities.** This assay was performed to assess the dUTPase activity in purified recombinant proteins. For details, see Methods and the text. 25 ng of each recombinant enzyme were incubated at 37°C for 30 min with dUTP-containing reaction buffer that was then terminated by heating for 3 min at 95°C. RLU- relative luminescence units.

All recombinant dUTPase versions were assayed for their enzymatic activity. We have employed a highly sensitive dUTPase assay- the commercially-available dUTPase assay (PPiLight Inorganic PPi assay, obtained from Lonza) - see Methods. This assay monitors the hydrolysis of dUTP to dUMP and PPi by a chain of reactions. In short, in the presence of AMP, the dUTPase-produced PPi is converted to ATP. Then, the newly-synthesized ATP is detected by the highly-sensitive assay of luciferase, which generates bioluminescence in the presence of luciferin. This produced bioluminescence is directly proportional to the amount of PPi present and, hence, to the initial dUTPase activity. The data shown in Figure 3 indicate that in this assay only the EIAV enzyme has a substantial dUTPase activity, while the other BIV dUTPase-derived proteins were practically inactive. Thus, with equal protein amounts, the ratios between the activities of EIAV enzyme and the BIV-derived enzymes is >95. In the specific assay, shown in Figure 3, we have used 25 ng of each protein. However, in case of the EIAV enzyme, activity could be detected even by using substantially-lower amounts of protein -as low as 1 ng (data not shown). To exclude the possibility that the assay conditions were not suitable for the BIV dUTPase, we have modified several components in the assay mixtures (see Methods). Thus, we have used HEPES buffer instead of Tris, used a wide ranges of MgCl_2_ concentrations (1–20 mM), varied the KCl from 12 up to 150 mM, DTT from 5 up to 20 mM and removed the BSA from the reactions. None of the various modified conditions could recover any significant traces of BIV dUTPase activity (data not shown). Collectively, it is quite clear that, under the *in vitro*-employed conditions (that are suitable for EIAV dUTPase), none of the three BIV-related putative dUTPase versions showed any enzymatic activity.

To be sure that the negative results for the BIV-derived dUTPases are not due to specific assay method employed, we have also used another method that takes advantage on the incorporation by Ex Taq polymerase of dUTP instead of dTTP in a PCR-based reaction. The dUTP-containing assay mixtures were first pre-incubated each with 25 ng of each dUTPase protein for varying periods, allowing dUTP hydrolysis. After heat inactivation, a PCR reaction was performed with the dUTPase products and the synthesized DNA was analyzed. In this assay, there is an inverse correlation between dUTPase levels and the extent of DNA synthesis. The results show again that while the recombinant EIAV dUTPase has a substantial dUTPase activity, none of the three BIV dUTPase-related proteins possess any detectable activity (Additional file 1: Figure S1). It should be noted that even much higher amounts (up to 250 ng) of the BIV-derived dUTPase proteins did not yield any detectable activity in this assay (data not shown).

### The dUTPase activity in viral extracts

**Figure 4:**
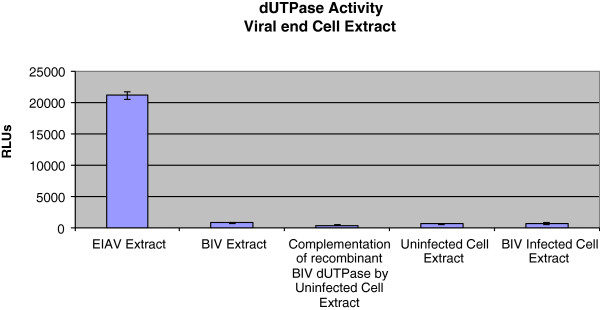
**The dUTPase activity in virions and in cell extracts.** The PPiLight pyrophosphate detection assay was performed to measure the dUTPase activity in viral extracts of WT BIV and EIAV, in Cf2Th cells that were chronically-infected by WT BIV (generating ~10^7^ viral particles/ml), or in uninfected Cf2Th cells. A dUTPase activity was also tested in a complementation assay of recombinant BIV dUTPase and the extract of uninfected Cf2Th cells. The assay conditions are described in Methods and in the text. All viruses and cells were lysed and then incubated at 37°C for 30 min with dUTP-containing reaction buffer. Viral extracts were prepared from ~8.8 × 10^7^ viral particles of either EIAV or WT BIV. Viruses were lysed using 0.5% Triton X-100 in the DMEM-FCS medium. Cell extracts were prepared from the pellets of ~5 × 10^4^ infected or uninfected Cf2Th cells by disruption in 100 μl of 0.5% Triton X-100 in PBS for 30 minutes on ice (followed by removing the insoluble material at 15000 rpm at 4°C). The activities in cells were then tested with equal amounts of the appropriate cell extracts. The complementation assay was performed by incubating 25 ng (in 5 μl) of the purified recombinant BIV dUTPase (the 74-residues version) with 10 μl of uninfected Cf2Th cells extract (prepared from ~5 × 10^4^ cells). All reactions were initiated by adding the 40 μl of dUTP-containing assay buffer for 30 min at 37°C, followed by heat inactivation, and then assayed as described in Methods. The shown values were obtained after subtracting the non-specific background produced with lysis buffer.

One possible explanation for the negative results, obtained for the recombinant BIV-related dUTPases, is that the design of the recombinantly-engineered proteins was wrong and, and contrary to the EIAV protein, maybe some important sequences are missing. Consequently, we have assayed the enzymatic activity in whole extracts of BIV virions. Viruses were generated from molecular clones of infectious viruses (see Methods). As a positive control, we have used EIAV virions. It is evident from Figure 4 that EIAV virions have indeed a substantial dUTPase activity. This result is in agreement with previous findings that non-primate lentiviruses package into their virions an enzymatically-active dUTPase [12, 13, 22]. However, the comparable extract of WT BIV yielded an insignificant dUTPase activity, barely above the background. In this specific experiment, the figure obtained for the EIAV extract was ~21000 RLUs and those for all other extracts (both BIV and cell extracts) were 400–700 RLUs. One explanation is that BIV does not package into virions the dUTPase-related protein or maybe some cellular factors are required to a dUTPase activity *in vitro*. To examine this possibility, we have also tested the dUTPase activity in extracts of cells chronically-infected with the WT virus. Again, the discerned activity, above the basal level of uninfected cells, was next to nothing. Another possibility for the lack of the *in vitro* dUTPase activity of BIV dUTPase is that cellular factors are required to complement this protein in order to recover dUTPase activity. Therefore, we also tested the complementation of the recombinant 74-residues BIV dUTPase by the extract of uninfected Cf2Th cells. The resulting data do not support this possibility (Figure 4). Taken together, no significant dUTPase activity is associated with BIV virions (or with the recombinant protein). It should be emphasized that the low levels of dUTPase activity in the extracts of both infected and uninfected cells apparently represent the normal basal cellular activity.

### The effects of mutations in the dUTPase-encoding gene on virus infectivity

Based on all results shown so far, the next question arising is whether the dUTPase-related segment is required at all for BIV infectivity. To this aim, we have generated two mutants of the infectious pBIV127 clone, where in both cases the dUTPase-encoding gene was modified. In the first clone, two amino acids, D48 and N57, were modified. The D48 residue, is equivalent to the active site D in homotrimeric dUTPases (within the third conserved motif, see Figure 1) that is responsible for activating the catalytic water molecule [39, 40]. Residue N57 was mutagenized, because it is located close to this motif and is conserved among all tested non- primate lentiviruses (Figure 1A). Thus, the double mutant, D48E/N57S, was generated (Figure 1B). In the second mutant BIV clone, 36 residues were removed from the dUTPase (amino acids 9–44); thus generating a truncated clone, designated Δ36 (Figure 1B).

**Figure 5:**
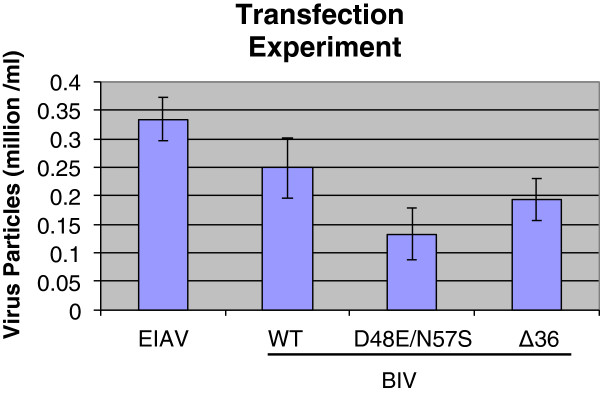
**The amounts of viral particles produced by transfection.** Wild-type and mutant BIV were produced by transfecting Cf2Th cells with the viral plasmids. The cells were treated with the following plasmids: pBIV127 (for WT virus), pBIV127 D48E/N57S or pBIV127 Δ36 (for the two BIV mutants), as described in Methods. In short, Cf2Th cells (70-80% confluent) were used for transfection by 4 ug DNA/well in 6 well plates with the TurboFect Transfection Reagent, following the recommended protocols. Cells were harvested 48 hours later and the number of viral particles was evaluated, as described in Methods. Representative data from three independent experiments are shown. The BIV virions are marked according to their dUTPase mutation.

BIV-susceptible Cf2Th cells (an adherent epithelial line, derived from normal fetal canine thymus) underwent transfections with the DNAs of the two mutant plasmids as well by the WT BIV- harboring pBIV127 plasmid. As a positive control, we have transfected the Cf2Th cells with the infectious EIAV plasmid, pSPEIAV19. All produced viruses were harvested and their copy number was evaluated by cDNA synthesis, followed by qPCR (Figure 5). The data show that the release of WT EIAV and BIV virions were comparable, while there was a minor decrease in producing the two BIV mutants. All transfection-produced virions were collected and equal viral particles were used to infect the susceptible naive Cf2Th cells (see Methods).

**Figure 6:**
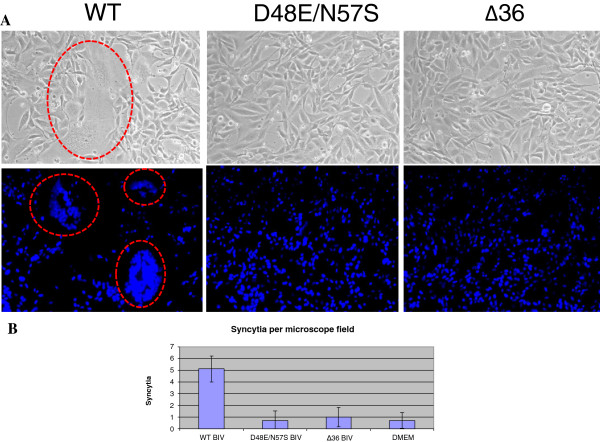
**The effects of infecting Cf2Th cells with the three different BIV versions.** The three BIV variants (WT and the two dUTPase mutants- D48E/N57S and Δ36) were used to infect the Cf2Th cells, as described in Methods. The infection of the cells by BIV was monitored by the formation of multi-cell syncytia after 10 passages. Since in Cf2Th cells, background syncytia of up to 10 nuclei per multinucleated cell could be occasionally observed, we have considered as BIV-specific only syncytia with more than 15 nuclei. **A**. The syncytia were visualized by phase contrast microscopy and by Hoechst dye for fluorescent staining of cell nuclei. The large, multinucleated giant cell syncytia are highlighted in red. **B**. A quantitative presentation of the average number of syncytia in a ×20 magnification microscopic field of the Cf2Th cells that were infected with WT and dUTPase mutated BIV. The control was performed with growth medium (marked- DMEM). Syncytia, obtained in three independent experiments and in 10 different microscopic fields from each, were counted.

**Figure 7:**
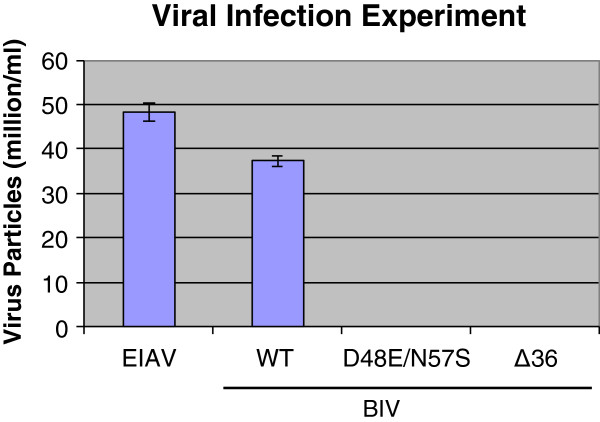
**The production of virions after infecting Cf2Th cells.** The cells that were 70-80% confluent were grown in 6 well plates and then infected with equal amounts (2.5 × 10^5^ viral particles per infection) of EIAV and of the different BIV variants (WT, D48E/N57S, Δ36)- all produced by transfection (see Figure 5). All infections were performed in the presence of 10 μg/ml DEAE-Dextran. The titers of the viral particles produced 4–6 weeks post-infection were determined, as described in Methods. The presented data were taken 5 weeks after infection (namely, 8 cell passages), as described in Methods.

Two methods were used to assess virus infectivity. The first was the formation of syncytia of the target cells, as it was already reported that BIV-infected Cf2Th cells undergo syncytia formations [41]. In contrast, EIAV did not induce in this cell line any formation of multinucleated cells (data not shown ), in accordance with previous data [42]. Therefore, we could not use this criterion for testing EIAV infectivity. The results presented in Figure 6 show that only WT BIV virions could induce considerable syncytia of the infected cells, even after two and a half weeks (five cell passages), while the two mutant BIV strains did not lead to any significant syncytia above background (even after 15 passages, namely 8 weeks of growth). The quantitative analysis of the produced multinucleated cells (Figure 6A) confirms this finding (Figure 6B). This finding suggests that the two mutant viruses were incapable of infecting the susceptible cells to any detectable levels. The lack of virus production by the two BIV mutant virions was further confirmed by the results shown in Figure 7. Here, we have used a second method for the evaluation of the *de novo*- synthesized and released viral particles. This highly-sensitive method involves the viral RNA reverse transcription into cDNA that is followed by qPCR technique (see Methods). As a positive control, we tested the infectivity of EIAV. Unlike both WT viruses, EIAV and BIV, the two dUTPase mutants of BIV did not produce any detectable amounts of newly-synthesized and released viral RNA. It should be also noted that the Cf2Th cells were found in our hands to be the most appropriate for producing infectious BIV particles by the WT pBIV127 plasmid, since the other cell lines tested (*e.g.,* 293 T and HeLa) produced after transfection virions that were incapable of infecting the Cf2Th cells (data not shown).

The lack of observed replication of the mutated BIV virions is not fully obvious. First, we could not detect in this study any dUTPase activity associated with even the WT protein. Second, the cells used were replicative Cf2Th cells. Therefore, even if a dUTPase activity would have been demonstrated, previous studies with other dUTPase-expressing lentiviruses imply that this enzymatically-active viral protein is required for viral replication in only non-dividing cells rather than in dividing cells [12–14].

### The presence of the RT, IN and dUTPase proteins in BIV virions

**Figure 8:**
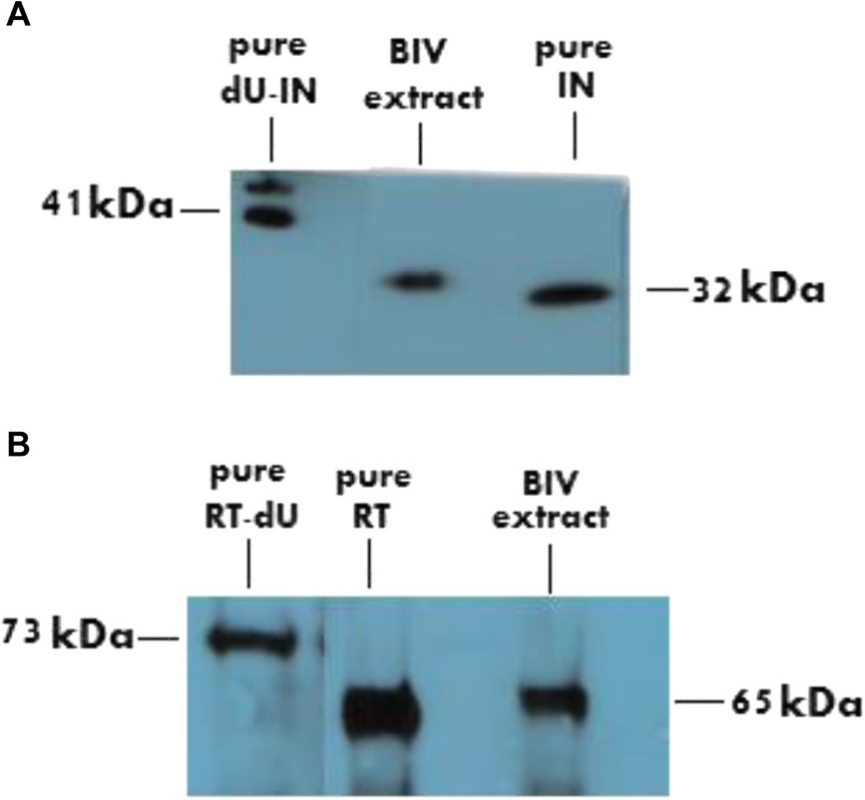
**Analysis of the RT and IN proteins present in BIV virions and the recombinant proteins.** BIV extracts and purified recombinant BIV proteins were prepared, as described in Materials. The proteins were separated by 10% SDS/PAGE and detected by western blot analysis. The blots were treated with 1:500 dilution of rabbit antiserum against the BIV IN-derived peptide **(A)** or antiserum against the RT-derived peptide **(B)**. After blocking the serum-treated blots, they were processed, as described in Figure 2 and in Methods.

Since the results presented so far imply that BIV requires for infectivity the complete dUTPase-related sequence, it was of interest to see whether the dUTPase peptide is fused to either RT or IN in the virions. To this aim, we tested the presence and size of RT and IN proteins in WT BIV virions by western analyses. This was done using antisera prepared against RT and IN-derived peptides (see Methods). As controls, we used the BIV-derived recombinant proteins versions, RT, RT-dU IN and dU-IN (Figure 8). Figure 8A shows the results obtained with the anti-IN serum. It is apparent that the size of the viral IN is close to that of the recombinantly-synthesized IN protein and it is definitely shorter than the recombinant dU-IN version. Likewise, Figure 8B (obtained with the anti-RT serum) shows that the viral RT is similar in size to that of the recombinant RT and, therefore, is not fused to the dUTPase. This information suggests that, during virus maturation, the viral protease cleaves the Pol polyprotein precursor near the sequences that were expected by us to generate the unfused final RT and IN proteins [20, 21]. However, after probing similar proteins blots (prepared from the WT BIV extracts) with the anti-dUTPase sera, no significant signals were detected (data not shown); although the same sera were efficient in detecting all recombinant proteins with dUTPase sequences (see Figure 2). These negative results were obtained independently with anti-sera prepared in two separate rabbits (data not shown). This lack of reactivity of the viral endogenous RT and IN proteins with the anti-dUTPase sera can lend further support for the absence of dUTPase-related sequences in these finally processed proteins. However, the free dUTPase segment was also not detected. This finding may result from either antibody titers, which are too low for detecting the viral dUTPase (although the recombinant proteins were easily detected- Figure 2), or from low undetectable levels of the dUTPase protein in the virions. Such a low level of the dUTPase 74-residues protein may result, by itself, from breakdown of the protein under the conditions used by us. This alternative is more likely than the option that the protein is not packaged into virions, as in all retroviruses the Gag-Pol precursor protein is first packaged in virions (as whole polyprotein) and only then matures by a restricted cleavage to the final protein products, which is performed by the viral protease [5]. It should be also mentioned that we have failed to detect the presence of the dUTPase protein in extracts of BIV-infected cells (using the same anti-dUTPase sera). On the other hand, purified dUTPase could be detected, even when mixed with cell lysates (data not shown). Hence, this issue regarding the presence of the dUTPase protein still needs to be further studied.

### The integration of cDNA from WT and mutant BIV into the genome of BIV-infected cells

**Figure 9:**
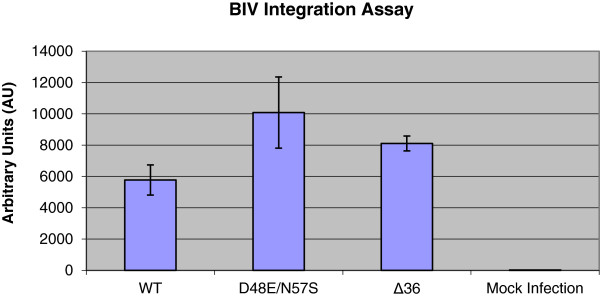
**The presence of WT and mutated BIV cDNAs in the genomic DNA of BIV-infected cells.** After transfecting Cf2Th cells by different proviral vectors, equal amounts of the newly-synthesized virions were used to infect Cf2Th cells. The genomic DNA of the infected cells was extracted and used as a template for PCR reaction using the repetitive canine primer, designated N201 [44] and an endogenous BIV primer (N142). Then, the amplified genome fragments were used as a template for qPCR reactions with the BIV-specific primers N142 and N143 (Table 1), in order to assess viral integration (WT and mutated BIV) into the cell genome. Uninfected cells genome was used as a mock control. For technical details, see Methods.

The importance of the dUTPase-related gene (or even the two active site related dUTPase residues D48 and N57) to BIV infectivity (Figures 7 and 8), raises the question at which step viral replication was blocked. To answer this, we have infected BIV-susceptible Cf2Th cells with equal BIV virion amounts of the WT, double mutant D48E/N57S or the Δ36 mutant, then harvested the cellular DNA and tested this DNA for the presence of integrated BIV cDNA. First, we have PCR-amplified DNA segments that span the cDNA-cellular DNA boundaries by using a known repetitive canine specific sequence, primer N201 [43, 44], and a BIV-specific primer (N142) (see Table 1). Then, reaction products were amplified by nested qPCR reactions that were carried out with internal BIV-specific primers (N142 and N143). The results shown in Figure 9 indicate that infection with the two mutant BIV strains did not cause any reduction in integration levels. To exclude the possibility that BIV cDNA integrations show some preference to cellular sequences in the proximity of the specific canine repetitive sequence N201 (Table 1), we have also performed the experiment with another known canine repetitive sequence, (designated N207, see Table 1) [43]. Here again, the levels of integrated cDNAs, synthesized by the two mutant BIVs, were not lower than WT cDNA (data not shown).

**Table 1:** **The synthetic oligonucleotides uses in this study**

Designations (lengths)	Sequences	Usage
N5A (36nt)	CCATGAGCCATATGGCCCCGTGTAGCCCTCCTGAGG	BIV dUTPase cloning
N5B (41nt)	CCATGCGAATTCAACAGGTCATGAAGACCTCATCTTTTTGG	BIV dUTPase cloning
N6A (33nt)	CCATGAGCCATATGCTAGCATACCAAGGCACAC	EIAV dUTPase cloning
N6B (32nt)	CCTAGCGAATTCCAGAATACTCCTGTACTTCC	EIAV dUTPase cloning
N110 (27nt)	ATCTCTCATGATCTTCAGTGGCTGAGG	BIV sequencing
N111 (26nt)	ATGCGGACATGAACAGTACCTAAGCC	PCR-based dUTPase assay
N120 (52nt)	GAGGTATTACCATGGTAGAGTCAGAGCTACAGCTACAGCTACTTAGCATAGG	BIV dUTPase point mutagenesis
N122 (26nt)	GAAGGCCATATGCATGGCTCTCTTGG	BIV dUTPase deletion mutagenesis
N123 (38nt)	GAATGTTACCATGGTAATGGCCTCAGGAGGGCTACACG	BIV dUTPase deletion mutagenesis
N124 (43nt)	GAATAGTCGACAGGAAATGCGTAGATTGAGGGTGCCTCTAAGG	BIV dUTPase point mutagenesis
N126 (28nt)	CACAATCCCAGGGAGTTGTAGAAAGAGC	BIV sequencing
N142 (20nt)	CCTTCAGCTTCCGTTCTTTC	BIV (qPCR) 93bp upstream to U3
N143 (19nt)	CGGAAGATGTCCCAAAGGA	BIV (qPCR) 33bp upstream to U3
N144 (20nt)	CTCTAATGGCCACTCAGCAA	MLV (qPCR)
N145 (20nt)	CCTCCCTGAGATCATCCTGT	MLV (qPCR)
N156 (23nt)	GACTCTTCTGTTGTATCGGGAAA	EIAV (qPCR)
N157 (18nt)	GGAAGCAAGGGGCTCAAG	EIAV (qPCR)
N162 (21nt)	TGCTGGTTTACCGGTTTATTG	Plasmid Backbone (qPCR)
N163 (20nt)	CAGGCATTTGAGAAGCACAC	Plasmid Backbone (qPCR)
N201 (24nt)	ATTCTAGAGGCCATTACGGCCTCG	Canine repetitive genomic sequence (PCR)
N207 (19nt)	GCCCGTCACCCATTCACCC	Canine repetitive genomic sequence (PCR)
Linker1-BIV (30nt)	TCGAGCACGTGTCTAGAGAATAATTGGGCC	Creating pET-22b(+)-*PmlI* plasmid
Linker2-BIV (22nt)	CAATTATTCTCTAGACACGTGC	Creating pET-22b(+)-*PmlI* plasmid

Taken together, the results presented in this manuscript emphasize the cardinal importance of the dUTPase-related gene region, and hence possibly the encoded protein segment, to BIV replication. We could not detect any dUTPase activity in both the recombinantly-expressed WT BIV dUTPase protein and in WT BIV virions. This finding is in contrast with the noticeable activity detected in the recombinant EIAV protein and in the EIAV particles. This result suggests that BIV is essentially different from EIAV (that served in this study as a dUTPase-positive prototype lentivirus) and, thus, from all other dUTPase-encoding lentiviruses. On the other hand, despite the lack of apparent enzymatic activity, mutating the BIV dUTPase-gene has led to a serious impairment of virus production. The behavior of BIV can be considered similar, at least in part, to that expected from abolishing the dUTPase enzyme in EIAV and other dUTPase-encoding lentiviruses. However, it was shown that active dUTPases are required in these viruses for infecting mostly non-replicating cells (and dividing cells can be easily infected by viruses with defective dUTPases). This outcome results most likely from the findings that the endogenous pools of dUTP are substantially lower in dividing cells (relative to the non-dividing ones) due to higher cellular dUTPases [12–14]. Yet, in the present case of BIV, all infectivity studies were performed with dividing Cf2Th cells; hence, under these conditions one could expect to see only a minor effect of the abolishment of the dUTPase activity. In an analogous case of a prokaryotic cellular dUTPase (from Mycobacterium), it was shown that this protein is essential for cell viability; yet the lack of dUTPase activity is not lethal [45]. To make things even more confusing, we could not even confirm that the mutated BIV enzyme lost its activity as a result of the introduced mutations, since no activity existed even in the WT protein. In all, the damage to virus production that resulted from modifying a gene encoding a seemingly-inactive enzyme (especially in the case of the double BIV dUTPase mutant D48E/N57S, where only two conserved residues were mutated) is puzzling.

So, what is the mode by which the mutations introduced into the dUTPase gene have led to blocking viral replication? The results presented in Figure 9 show that the extent of viral cDNA synthesis and the subsequent cDNA integration into the cellular DNA were not affected by the dUTPase mutations, indicating that the early steps of the retroviral infection (*i.e.,* cell entry, reverse transcription and integration) were quite normal. Since these steps are known to be affected by an aberrant proteolytic processing of the viral Gag-Pol polyprotein precursor [5], it is extremely unlikely that two studied dUTPase mutants could affect this processing. Altogether, the probable explanation is that either the integrated DNA of the mutant virions is defective (due to possible multiple mutations introduced during reverse transcription) or that the original dUTPase mutations have led to blocks in viral replications at steps post integration (*e.g*., transcription, protein synthesis assembly and budding). At this stage, it seems to us that the first alternative may be more likely. This supposition is based on the finding that we could get newly-synthesized virions by transfecting cells with the complete, albeit mutated, BIV plasmids, a process that bypasses the normal retroviral reverse transcription and integration steps (Figure 5). Interestingly, BIV has probably the most complex genome of the non-primate lentiviruses [32]. Therefore, another potential explanation for the enigma surrounding BIV dUTPase could be that the DNA sequences in the dUTPase-encoding gene of BIV are important for viral infectivity, because they may overlap parts of the alternatively-spliced genes that encode the small regulatory viral protein. Nonetheless, the presently-predicted genetic map of BIV implies that none of these regulatory/accessory genes of BIV (*i.e., vif*, *tat*, *rev*, *vpy, vpw*, and *tmx*) can overlap the dUTPase gene [32]. Further biochemical research will be required to substantiate this conclusion. Alternatively, it might be possible that the dUTPase sequence interacts with other viral and/or cellular factors that participate in the viral replication cycle. These outlined alternatives and questions lead the way for future studies to elucidate the molecular mechanisms underlying the importance of the dUTPase-related gene to BIV infectivity.

## Conclusions

Non-primate lentiviruses express a dUTPase enzyme that is important for viral growth in non-replicating cells. In BIV, the dUTPase-expressing gene encodes a putative protein that is smaller by about half than all known lentiviral dUTPases. We show herein that, as could be expected from the presence of a truncated dUTPase, the recombinant BIV protein is devoid of any dUTPase activity. Accordingly, unlike other non-primate lentiviruses, no dUTPase activity was also detected in BIV virions. Surprisingly, a modification of the dUTPase gene, leading to the change of merely two residues at the putative catalytic site and at vicinity, resulted in totally-defective viruses, although both viral cDNA synthesis and integration were apparently unharmed. These results emphasize the critical importance of the dUTPase-related sequence to BIV replication, despite the lack of any catalytic activity, and raise the possibility that functions, additional to dUTPase activity *per se*, are also critical to lentiviral replication.

## Methods

### Synthetic oligonucleotides

All oligonucleotides used in this study are described in Table 1.

### Plasmids

#### BIV and EIAV viral plasmids

1. BIV plasmid: designated pBIV127, which contains the complete infectious proviral DNA clone of BIV [46] was already used by us in the studies of BIV RT and IN enzymes [20, 21].
2. EIAV plasmid: designated pSPEIAV19, contained the entire infectious EIAV proviral DNA [47]. Both viral plasmids were generously provided by the “NIH AIDS Research & Reference Reagent Program”.

#### Other plasmids

The pET22b(+)- vector (from Novagen) was used for subcloning of the BIV dUTPase fragment employed for mutagenesis of the protein (see below).

#### Bacterial expression plasmid

The pT5M-NdeI-6His plasmid, which is a derivative of the pT5-NcoI-6His plasmid that was used frequently by us [20, 21, 33, 37, 48, 49], was generated by replacing the *NcoI* site with a *NdeI* (both containing the initiation ATG). This plasmid adds the sequence MHHHHHH- to the N-terminus of the expressed proteins.

### Proteins

Recombinant BIV RT-dU and BIV dU-IN were expressed and purified as described in detail earlier by us [20, 21].

### Antibodies

The commercial antibodies used were: monoclonal mouse anti-6HIS horseradish peroxidase (HRP) - conjugated antibody (from Sigma) and donkey HRP-conjugated anti-rabbit IgG antibody (purchased from abcam). The anti-peptide antibodies were prepared in rabbits against synthetic peptides derived from the BIV dUTPase, RT and IN proteins. All peptides and the antisera were prepared by Genemed Synthesis. The dUTPase-derived peptide has the sequence CDSELQLQLLNIGTEHIRIQK, the IN-derived peptide is CVERAHRDLKDRLAAY and the RT- derived peptide is NRDDHKQIVQEIRDKLGSC.

### Bacteria

#### BL21(DE3)pLysS

This *E. coli* strain was used for expressing the recombinant proteins.

#### DH5α

This *E. coli* strain was used for standard cloning applications and plasmid amplifications.

### Cell lines

#### Cf2Th cells

This adherent epithelial cell line (kindly provided by Prof. L. Sherman) is derived from normal fetal canine thymus and was found to be susceptible to transfection, infection and proliferation by BIV and EIAV [41, 50]. These replicating cells were grown in Dulbecco’s modified eagle’s medium (DMEM) supplemented with 10*%* heat-inactivated fetal calf serum (FCS), 2% L-glutamine and 1% penicillin-streptomycin antibiotics. Cells were maintained at 37°C in a humidified atmosphere of 95% air and 5% CO_2_.

### Construction of the dUTPase-expressing vectors

The fragments of EIAV dUTPase and BIV dUTPase were first synthesized by PCR, using primer sets N5A/N5B and pBIV127 (for BIV dUTPase) or N6A/N6B and pSPEIAV19 (for the EIAV protein). All primers are described in Table 1. Reactions were carried with 200 μM of each dNTP and 2.5 units of Ex Taq polymerase (TaKaRa). They were first incubated at 95°C for 4 min following this procedure: denaturation at 95°C for 30 sec, annealing at 55°C for 30 sec and elongation at 72°C for 60 sec. This was done for 28 consecutive cycles. The resulting dUTPase-encoding genes were designed to include a *NdeI* at the 5′ end and an *EcoRI* site after the terminator codon. These DNAs were digested with *Nde*I and *EcoRI*, and then ligated into the *Nde*I and *EcoRI-*cleaved pT5M-*NdeI*-6His expression plasmid (see above). The designations of the created plasmids were: pT5-6His-BIV-dUTPase and pT5-6His-EIAV-dUTPase (for BIV dUTPase and EIAV dUTPase, respectively). Since the putative sequence of viral BIV dUTPase (see Figure 1) starts with PCSPPE-, the final amino terminus of this protein is: MHHHHHHM-PCSPPE-, *i. e.,* 8 extra residues were added to the authentic viral protein. The EIAV protein starts with MLAYQG-; therefore, the final N-terminus is MHHHHHH-MLAYQG- (7 residues added, since the authentic proteins starts with a Met). The authenticity of the inserted fragment was confirmed by sequencing the cloned genes.

### Recombinant expression and detection of the BIV and EIAV-derived dUTPases

*Escherichia coli* BL21(DE3) cells were transformed by the newly-generated expression plasmids, pT5-BIV-dUTPase for wild-type (WT) BIV dUTPase and pT5-EIAV-dUTPase for WT EIAV dUTPase. The proteins were expressed as follows: a single-transformed colony was grown in 10 ml of LB medium, containing 100 μg/ml ampicillin, at 37°C with shaking at 250 rpm. After overnight growth, 500 μl of culture was transferred to 2 L of fresh LB medium for growth at 37°C. At OD_600_ = ~0.6 IPTG was added to a final concentration of 1 mM. The culture was further grown for additional 4 hours at 37°C. Aliquots of the induced bacteria were removed and their total protein content was analyzed by sodium dodecyl sulfate polyacrylamide gel electrophoresis (SDS-PAGE). Since all expressed proteins contained the six-histidine sequence, protein expressions were confirmed by SDS PAGE of the bacterial extracts that were followed by western analyses, using monoclonal anti-6HIS HRP conjugated antibody (Sigma).

### Purification of the EIAV and BIV dUTPases

#### Cell lysis

The cells from the induced medium were harvested by centrifugation at 8000 rpm for 5 min (in a Fiberlite F13-14X50cg rotor in a Sorvall RC6 Plus centrifuge). Cell lysates were prepared from the cell pellets from 2 L cultures by suspending the cells into 30 ml of lysis buffer - 50 mM Tris–HCl pH 8.0, 50 mM NaCl, 1 mM EDTA, 10% glycerol, 10% sucrose, 5 mM MgCl_2_, 1 mM DTT, 1 mM phenylmethylsulfonyl fluoride (PMSF), 0.5% Triton X-100, 1 mg/ml lysozyme and 10 μg/ml DNase I (Roche). After adding this buffer, the mixture was stirred at 4°C for 30 min and then underwent sonication. The lysed bacterial suspensions were centrifuged at 13,000 rpm for 30 min at 4°C. The soluble clear fractions were collected and the proteins were analyzed by SDS-PAGE and by western blotting, using monoclonal anti-6HIS HRP-conjugated antibody (data not shown).

#### Protein purification

The bacteria lysates, containing different dUTPases, were loaded onto a Ni-NTA Agarose columns (Qiagen) (2 ml of Ni-NTA) that were pre-equilibrated with the washing buffer: 50 mM NaH_2_PO_4_, 150 mM NaCl, 5 mM imidazole, final pH 8. The columns were then washed with 50 ml of this washing buffer. Finally, elution was performed with 20–500 mM imidazole linear gradients in the same buffer [37, 48, 49, 51]. The purity of the column-eluted dUTPase enzymes were assessed by SDS-PAGE and western analysis with monoclonal anti-6HIS HRP- conjugated antibodies (from Sigma). Protein concentrations were determined by the Bradford method, using BSA as a standard. After protein analyses, all pooled protein fractions were aliquoted and stored at -80°C.

### Enzymatic assay for detecting dUTPase activities

We have assessed the dUTPase-dependent production of inorganic pyrophosphate (PPi) by a chain of reactions. This method was used to assay the dUTPase activity in both the purified recombinant proteins as well as in BIV and EIAV virions. The dUTPase-produced PPi was monitored with the highly-sensitive non-radioactive bioluminescent assay, the “PPiLight Pyrophosphate Detection Kit” (from Lonza, Rockland, USA). The viral dUTPase hydrolyzes dUTP to dUMP and PPi. The detection reagent catalyzes the conversion of AMP and the enzymatically-produced PPi to ATP. Then, the assay uses luciferase, which produces light from the newly formed ATP and luciferin. The initial dUTPase reactions were performed in 40 μl volumes containing 50 mM Tris–HCl, pH 7.5, 20 mM MgCl_2_, 20 mM KCl, 5 mM DTT, and 0.1 mg/ml BSA with 100 μM high purity dUTP. To this mixture, 1 ng to 25 ng of the purified dUTPase proteins or 3.5 × 10^6^ - 8.8 × 10^7^ lysed BIV or EIAV viral particles were added. This was followed by initial incubations at 37°C for 30 min followed by treatments for 3 min at 95°C. After this initial dUTPase reactions, 20 μl of PPiLight converting reagent were added to the whole 40 μl of the dUTPase reaction products in black wall 96 well plates (Corning). The mixtures were incubated at room temperature for 30 min; then 20 μl of PPiLight detection reagent were added and incubated for 30 min. Finally, luminescence was measured using Mithras LB 940 Multimode Microplate Reader (Berthold Technologies). In all experiments, the produced relative luminescence units (RLUs) are directly proportional to concentrations of the dUTPase-produced PPi.

### Mutagenesis of the infectious BIV-containing plasmid

For these studies, the pET-22b (+) plasmid was modified by adding a synthetic DNA linker that adds the *PmlI* restriction site. This linker is composed of the two synthetic oligos, designated linker1-BIV and linker2-BIV (see in Table 1). The formed duplex, which has pre-formed *ApaI* and *XhoI* sites was ligated into the *ApaI* and *XhoI* pre-digested pET-22b(+) plasmid; thus generating the pET-22b(+)-*PmlI* plasmid. The pBIV127 DNA was digested with *ApaI* and *PmlI* and a 3813-bp fragment, containing the putative dUTPase sequence, was isolated and subcloned into the *ApaI and PmlI*- predigested pET-22b(+)-*PmlI*, thus creating the pET-22b(+)-WT dUTPase vector.

The mutated dUTPase genes were first subcloned into pET-22b(+)-WT dUTPase plasmid. To create the double mutation (D48E/N57S) and a deletion mutation (Δ36) in the dUTPase gene, parts of the WT sequence were amplified by PCR using N120/N124 and N122/N123 mutagenesis primers, for the double mutant and the deletion mutant, respectively. Subsequently, the generated double mutant gene was digested by *NcoI* and *SalI* and subcloned into the *NcoI* and *SalI* pre-digested pET-22b(+)-WT dUTPase vector (generating the pET-22b(+)-D48E/N57S dUTPase vector). Similarly, the deletion mutant was created by *NcoI* and *SalI* digestion following ligation into the *NcoI* and *SalI* pre-digested pET-22b(+)-WT dUTPase vector (generating the pET-22b(+)-Δ36 dUTPase vector). The validity of the subcloned amplified dUTPase genes was confirmed by DNA sequencing.

To introduce the mutated dUTPase-encoding DNA fragments back into the provirus pBIV127, the two mutated pET-22b(+)-dUTPase DNA clones were digested with *ApaI* and *PmlI* and ligated into the *ApaI* and *PmlI* pre-digested proviral pBIV127 vector. The final proviral BIV vectors were designated pBIV127 D48E/N57S (for the dUTPase double mutant plasmid) and pBIV127 Δ36 (for the Δ36 deletion mutant). The modified dUTPase-encoding sequences were confirmed by DNA sequencing.

### DNA transfections and generation of wild-type and mutant BIV and EIAV

WT pBIV127 and the two mutated BIV proviral plasmids, as well as pSPEIAV19 (the WT EIAV proviral plasmid) were used to transfect Cf2Th cells, employing the TurboFect Cell Transfection Reagent (from Fermentas). According to the manufacturer’s instructions, 4 μg of each DNA were mixed with 6 μl of TurboFect reagent in a final volume 400 μl of DMEM-FCS. The ~80% confluent Cf2Th cells in 6 well plates were supplemented with 4 ml of DMEM-FCS complete medium and incubated with the TurboFect -DNA mixture for 18 hours. The medium was then removed and cells were washed twice with 5 ml of phosphate-buffered saline (PBS). To check virus titers, the transfected cells were cultured in DMEM-FCS medium for 24, 48, 72, 96 or 120 hours. At each time point, the medium was removed, centrifuged for 60 min 5000 g at 4°C, filtered through a 0.45 μM Millipore filter and HEPES buffer (pH 7.6) was added to a final concentration of 20 mM. The virus-containing medium was frozen in liquid nitrogen and stored at -80°C. The number of BIV or EIAV particles in the medium was analyzed afterwards, as described below.

### BIV and EIAV infection of cells

About 80% confluent Cf2Th cells in 100 mm plates were incubated with BIV or EIAV virions (WT and mutants) for 6 hours in the presence of DEAE-Dextran (10 μg/ml). The cells were incubated at 37°C to permit virus adsorption. Then, to remove unbound virus and traces of provirus plasmids, washing were performed three times with 5 ml PBS. Cells were then supplemented with 10 ml of DMEM-FCS medium. Mock-infected cells were treated similarly but with no virus. Next, the BIV-infected cells were sub-cultured into a new 100 mm plate at 1:3 split for 6–12 passages (done every 3–4 days). Every passage, the cells were re-fed by removing two-thirds of the supernatant and replacing it with fresh medium, while being monitored visually for the formation of syncytia. At specified times, supernatants were removed, centrifuged for 60 min 5000 g at 4°C, and filtered through a 0.45 μM Millipore filter and 20 mM HEPES buffer (pH 7.6) was added. Subsequently, the virus-containing medium was frozen in liquid nitrogen and stored at -80°C. The numbers of viral particles in the medium were analyzed afterwards, using the combination of reverse transcribed viral RNA that was followed by the qPCR of the cDNA products, as described below.

### Preparation of BIV or EIAV virions

For each round of ultracentrifugation, 30 ml of viral supernatant was centrifuged at 20,000 rpm for 90 minutes at 4°C in a Beckman SW28 swinging bucket rotor in over 20% sucrose “cushions” in TNE buffer (50 mM Tris–HCl, pH 7.4, 100 mM NaCl and 0.1 mM EDTA). The pellets were gently re-suspended in 200 μl DMEM-FCS medium (supplemented with 20 mM HEPES, pH 7.6). The concentrated virions were then stored at -80°C.

### Viral RNA purification and qPCR analysis

Frozen 200 μl BIV or EIAV virus samples, which were collected after transfection or infection, were used to compare viral particle productions. A constant amount of 5 μl virions of murine leukemia virus (MLV) (~2 × 10^7^ virus particles/ml, calculated by qPCR assay and standard curve analysis) was added to each sample to serve as internal efficiency controls for the viral RNA purification and cDNA synthesis steps. The presence of MLV cDNA was monitored using primers N145 and N145 (Table 1). Total viral RNA was extracted from the medium using a “High Pure Viral RNA Kit” (from Roche). The detailed extraction and elution procedures are described in the Roche products instruction manual. Prior and post RNA extraction, the samples were treated with RNase-free DNase I (Fermentas) to eliminate any provirus-containing plasmid contaminations. After this DNase I digestion, the enzyme was inactivated by incubation at 65°C for 10 min. 3 μl of DNase I-treated viral RNA was used as a template for the syntheses of viral cDNAs, as described in Roche product instruction manual (Transcriptor Universal cDNA Master).

The synthesized viral cDNAs solutions were diluted in ultra-pure nuclease free water (at the ratio of 1:50) and used as a template for qPCR reactions (with FastStart SYBR Green Master Roche). The qPCR reactions were performed in 96-well blocks with Applied Biosystems StepOnePlus™ Real-Time PCR Systems using FastStart SYBR Green Master (Roche). The sequences of virus-specific primer sets that were chosen to quantify total number of viral particles are shown in Table 1. A set of plasmid (pBIV127) backbone-specific primers (oligos N162 and N163) was used as controls to exclude possible plasmid contaminations in the obtained cDNA samples. For each primer set, an appropriate provirus plasmid was chosen to create a 10-log-fold standard curve for direct quantification of all samples. Based on these standard curved, the amounts of viral particles were calculated. Each qPCR contained 10 μl of 2XFastStart SYBR Green Master mix, 4 μl of H_2_O, 4 μl of a 2 pmol/μl of the specific primer mix for each BIV sample (N142 and N143, derived from the sequence located 93–33 nts upstream to the U3, see Table 1) and 2 μl of diluted viral cDNA. The cycling conditions were as follows: 95°C for 10 min; and 40 cycles of 95°C for 10 sec and 59°C for 30 sec. At each cycle, accumulation of PCR products was detected by following the increase in fluorescence of the reporter dye, double-stranded DNA-binding SYBR Green. Following amplification, melting temperature analysis of PCR 60-nts long products was performed to determine the specificity of the PCR. Melting curves were obtained from 55-90°C, with continuous fluorescence measurements obtained at every 1°C increase in temperature. All reactions were carried out in triplicates and a non-template control was performed in every analysis. In addition, the concentration of each virus preparation was calculated for each reaction, and the mean, standard deviation and statistical significance were determined.

### Analyses of integrated cDNA into the cellular genomic DNA

The WT pBIV127 and the two mutated BIV proviral plasmids were used to transfect Cf2Th cells, as described above in the Methods section. After the transfection, cells were washed and incubated for another 48 hours to allow viruses production. Equal amounts of ~5 × 10^5^ copies of the generated virions were used to infect 80% confluent Cf2Th cells in 35 mm plates. Naïve cells were incubated with 2 ml BIV-containing medium (WT or mutants) for overnight in the presence of 10 μg/ml of DEAE-Dextran. After incubation, the infected cells were washed, harvested and the whole cellular genome was extracted, using the “Quick-gDNA MiniPrep” (ZYMO RESEARCH). Aliquots of these DNA preparations (2 μl) were amplified in PCR reactions with Ex Taq polymerase (TaKaRa) employing the N142 and N201 primers (95°C at 4 min, followed by 35 cycles of - 95°C at 30 sec, 55°C for 30 sec, 72°C for 8 min). Since the N201 primer is based on repetitive elements in canine genome [44] and the N142 is an endogenous BIV primer, this PCR reaction amplified DNA segments spanning BIV cDNA-cellular DNA. The resulting PCR products were diluted in ultra-pure nuclease free-water (at the ratio of 1:100) and used as a template for nested qPCR reactions, using the BIV-derived primers N142 and N143, as described above. As mentioned above, we have also tested each cDNA preparation by separate qPCR reactions with plasmid backbone-specific primers (oligos N162 N163) to exclude any possible minor plasmid contaminations.

## Electronic supplementary material

Additional file 1: Figure S1: The PCR-based dUTPase activity assay of the different recombinant and purified dUTPases. (DOCX 148 KB)

## Abbreviations

BIV: bovine immunodeficiency virus
EIAV: equine infectious anemia virus
CAEV: caprine arthritis-encephalitis virus (CAEV)
JDV: Jembrana disease virus
MMTV: mouse mammary tumor virus
dUTPases: deoxyuridine 5′-triphosphate nucleotide-hydrolases
PPi: pyrophosphate
RT: reverse transcriptase
IN: integrase
PR: protease
RLUs: relative luminescence units
HRP: horseradish peroxidase
qPCR: quantitative PCR.
