# Supplementary material for: The dUTPase-related gene of bovine immunodeficiency virus is critical for viral replication, despite the lack of dUTPase activity of the encoded protein
Source: Retrovirology. 2014 Aug 12;11:60. doi: 10.1186/1742-4690-11-60 (PMC4261571; doi:10.1186/1742-4690-11-60)
Supplement: Supplementary file 1 — Additional file 1: Figure S1: The PCR-based dUTPase activity assay of the different recombinant and purified dUTPases. (DOCX 148 KB) [file 12977_2014_3939_MOESM1_ESM.docx]

**Supporting Data**

**The dUTPase-related gene of bovine immunodeficiency virus is critical for viral replication, despite the lack of dUTPase activity of the encoded protein (by- N. Voronin, E. Herzig and A. Hizi).**


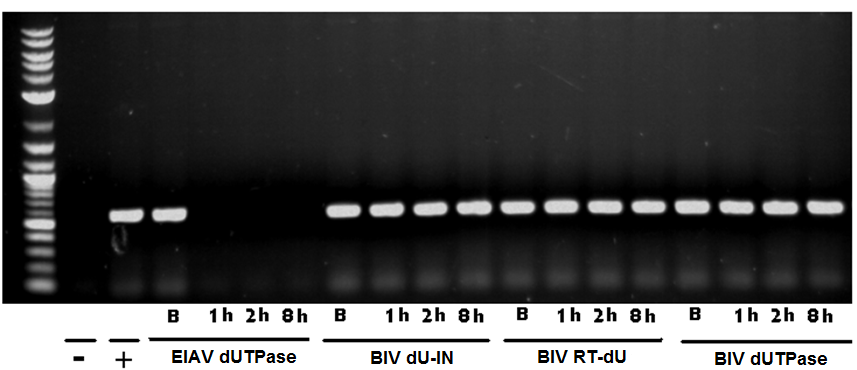


**Figure S1: The PCR-based dUTPase activity assay of the different recombinant and purified dUTPases.**

25 ng of each of the purified recombinant enzymes (*i., e.,* WT BIV dUTPase, BIV RT-dU and BIV dU-IN and WT EIAV dUTPase) were pre-incubated at 37ºC for different periods of times in 55 µl reaction buffer, containing 50 mM Tris-HCl pH 7.5, 20 mM MgCl_2_, 20 mM KCl, 5 mM DTT, 0.1 mg/ml BSA and 3 mM high purity dUTP (Fermentas). Then, the reactions were terminated at 98ºC for 3 min. The dUTPase activity was assessed by testing the residual dUTP levels, based on dUTP incorporation into DNA (instead of dTTP) in a PCR-based reaction. After a 1:4 dilution of the initial dUTPase reaction mixtures, DNA was synthesized at final concentrations of 150µM of each of the three dNTPs-dATP, dCTP and dGTP. These PCR reactions were performed with Ex Taq polymerase (TaKaRa), using a provirus pBIV127 plasmid as a template and the N111/N126 primers (Table 1), leading the synthesis of 607bp products. These DNA products were analyzed by electrophoresis a 1% agarose gel.

Shown on the left is a DNA marker that is a 100bp ladder. B, assayed with heat-inactivated dUTPase (for 3 minutes at 95ºC). The control reactions (shown on lanes 2 and 3 from the left) were carried out with no dUTPase: (-) with no dUTP present, (+) with dUTP.
